# The Boarding-Out of Pauper Children

**Published:** 1905-08-05

**Authors:** 


### THE BOARDING-OUT OF PAUPER CHILDREN.

The St. Pancras Guardians resolved some time ago to abandon the system of boarding-out the children under their control, "seeing that admirable schools had been erected." In this step St. Pancras is only following the example of other metropolitan boards of guardians. The resolution is quite comprehensible, though there may be reasons for regretting it. No one doubts that, properly carried out, boarding-out is the best system for bringing up such children as fall under the care of the poor-law. The home and its conditions are as like as may be to a home of the class to which the child by birth belongs, and the children associate freely at school and at play with ordinary children and so lose the idea of belonging to a class apart. If they are paupers, at least they need not know it, and thus they escape that habit of dependence on outside aid which brings so many poor-law children back to ask help from the guardians at every crisis of their adult life. There is so much to be said in favour of boarding-out, that it will be difficult for many people to understand why there is now a reaction against it, especially in large unions. But it is a system which demands endless care and watchfulness. The number of absolutely suitable homes is far more limited than the number of children who require them. Frequent surprise visits are necessary, nor will it do to trust to the supervision exercised by the local committee, however well-meaning the members of it may be. Miss Mason, the Local Government Board Inspector of Boarded-Out Children, who believes thoroughly in the excellence of "the boarding-out system, if accompanied by supervision which is both thorough and adequate," has pointed out the difficulties which surround it. She says: "I have found some of the best and worst homes together at the same time, under the same committee. This shows that what is satisfactory is due to the kindness of particular foster-parents, not to the selection and supervision of the homes by the committees." Unfortunately many members of these local committees regard the system rather as a means of providing for their own dependents than as a method of bringing up the children. "One gentleman recommended a disabled coachman and his wife, who received 32s. a week (besides extras) for eight children." In many cases the rates of other places must have been used to relieve the local rates by providing in this fashion for old people who would otherwise have needed help from the latter. Facts like these have prejudiced people against the entire system. It cannot be said, however, that the opponents of the system have been characterised by consistency. The Rev. T. W. Fowle, who contributed the volume on the Poor-law to the English Citizen Series quoted as a condemnation of the boarding-out system a sentence from the report of the Secretary of State for Victoria (where boarding out had been adopted) which said: "A majority of the children are probably much better cared for under the boarding-out system than children of the same class by their own parents." He depicted this as the compelling of respectable families to contribute to the income of persons of their own class (but probably better off since they had no dependent children) by letting them have the care of the children of their own class, but disreputable and pauperised. But as these same persons must contribute to the maintenance of such children in schools, the appeal to personal jealousy should have but little weight. Nothing is saved, indeed children cost more in poor-law schools than when boarded out. On the other hand, Mrs. Idris, a member of the St. Pancras Guardians, supports the abandonment of boarding-out on the ground that the children are brought up to drudgery by their foster-parents. Probably both these opinions are true in different cases, but as criticisms they both miss the mark. The foster-parents are but a means to an end; that end the bringing up of children so that they shall not in adult life continue to be paupers. Which system, the school or the boarding out, best develops those qualities of energy and self-reliance on which depend the future welfare of the children? At present boarding-out is adopted or abandoned for reasons which are either sordidly selfish or sentimentally unpractical, and in either case, short-sighted. Before coming to a decision on the merits or demerits of the system, one would want full statistics as to the ultimate results of both methods of treatment—these statistics, not to comprise merely the health, weight, and intellectual advancement of the children during their years of pupilage, but the positions they have achieved in life, and whether or not they have returned, or are likely to return to the condition of pauperism in which they were brought up, and from which it must be the desire and effort of the guardians permanently to uplift them.

